# Health workers’ strikes: a plea for multisectoral action

**DOI:** 10.2471/BLT.19.238279

**Published:** 2019-07-01

**Authors:** Peter Salama, Michelle McIsaac, James Campbell

**Affiliations:** a Universal Health Coverage Across the Life Course Division, World Health Organization, Geneva, Switzerland.; b Health Workforce Department, World Health Organization, avenue Appia 20, 1211 Geneva 27, Switzerland.

Health workers’ strikes are of increasing concern. In this issue of the *Bulletin*, we present research covering the period 2009–2018 across a group of 31 low-income countries.^1^ Many people who live in low-income countries lack access to quality health care, and interruptions in services affect some of the most vulnerable populations.^2^ In the 10-year period studied, an average of one out of every three days was affected by disruption in care attributable to health workers’ strikes. In 2018, the total number of days of work stoppage observed across all low-income countries was 170; care was disrupted, on average, every other day, as opposed to one of every three days across the 10-year period.^1^

Wage concerns are detailed in more than half of the observed health worker strikes in low-income countries. A quarter of these cases also raised concern regarding delayed salary payments. Working conditions and governance were each raised in about a third of the strikes observed. In addition, two strikes were attributed in part to direct safety concerns.^1^ This wide range of concerns undermines the perception that strikes are only related to collective bargaining for improved pay.

The effects of health workers’ strikes have far-reaching consequences, not only in low-resource settings,^1^ but also in settings affected by fragility, conflict and violence. Such settings are vulnerable to epidemics and pandemics and have a disproportionate burden of noncommunicable disease.^3^ Currently, there are 35 such settings globally^4^ with around half of these in low-income countries.

In 2018, 161 attacks targeted health workers, with more than half of all attacks occurring in low-income countries; attacks are even more prevalent in settings affected by fragility, conflict and violence.^5^ Polio vaccination workers were targeted and killed in Pakistan, and doctors killed in the Libyan conflict.^6^ Health workers providing care during the Ebola disease outbreak in the Democratic Republic of the Congo threatened to strike due to attacks and targeted violence.^7^ One of these attacks resulted in the death of World Health Organization epidemiologist Richard Valery Mouzoko Kiboung.^6^

Health workers’ strikes are not an issue isolated to low-resourced countries. In the first months of 2019, there were reports of health workers’ strikes in France, India, Ireland, Israel, Kenya, New Zealand, Nigeria, United States of America and Zimbabwe. Health workers’ strikes are a global issue of growing significance, particularly as the global community focuses on universal health coverage (UHC) and leaving no one behind.

However, solutions are available. The health sector needs to work with counterparts across other sectors to address the contextual and upstream factors associated with health workers’ strikes. Heads of State can enable multisectoral action, with political leadership across government ministries, to resolve these challenges, caused by factors across multiple sectors. Multisectoral action is central to both limiting the detrimental impact on health systems and populations, as well as creating an enabling, safe and effective working environment for health workers, thus reducing the likelihood of future strike action.

Investing in decent working conditions for health workers is a vital step to ensure the existing investments in the human capital of health workers are used efficiently. Multilateral organizations need to come together to ensure the protection and security of all health workers and health facilities in all settings. Policy-makers need to promote intersectoral collaboration at the national, regional and international levels, and engage civil society, unions and other health workers’ organizations along with the private sector.

The upcoming United Nations high-level meeting on UHC in September 2019 is an opportunity to acknowledge the importance of decent work^8^ for health workers and highlight the need for actions across government sectors. For example, deteriorating working conditions can be linked to weak labour regulations and the terms and conditions of employment, many of which are determined outside of the health ministry. Delayed or irregular payment of salaries are linked to the structure and sources of salary, as well as governance of payment mechanisms. Adequacy of pay, particularly for public-sector employees, is often linked to national macroeconomic and financial constraints. Safety concerns and attacks on health workers and health facilities are generally symptomatic of wider national or local security risks.

When health workers’ strikes take place, the rights, roles and responsibilities of health workers are often discussed, with many questioning the ethics of strikes in the health sector. Consideration of the roles and responsibilities of the national, regional and international partners in addressing the underlying issues must be added to the discussion. If we are to make progress on UHC there must also be broad intersectoral action on effective dispute prevention and investment in health workers, as outlined in the High-Level Commission for Health Employment and Economic Growth.^9^
